# An adaptive gBOIN design with shrinkage boundaries for phase I dose-finding trials

**DOI:** 10.1186/s12874-021-01455-y

**Published:** 2021-12-13

**Authors:** Rongji Mu, Zongliang Hu, Guoying Xu, Haitao Pan

**Affiliations:** 1 Clinical Research Center, Shanghai Jiao Tong University School of Medicine, Shanghai, 200025 China; 2 College of Mathematics and Statistics, Shenzhen University, Shenzhen, 518060 China; 3 Jiangsu Hengrui Medicine Co., Ltd, Shanghai, 201203 China; 4 Department of Biostatistics, St. Jude Children’s Research Hospital, Memphis, 38105 TN USA

**Keywords:** Bayesian adaptive design, Phase I dose-finding trial, Shrinkage boundaries, Maximum tolerated dose

## Abstract

**Background:**

With the emergence of molecularly targeted agents and immunotherapies, the landscape of phase I trials in oncology has been changed. Though these new therapeutic agents are very likely induce multiple low- or moderate-grade toxicities instead of DLT, most of the existing phase I trial designs account for the binary toxicity outcomes. Motivated by a pediatric phase I trial of solid tumor with a continuous outcome, we propose an adaptive generalized Bayesian optimal interval design with shrinkage boundaries, gBOINS, which can account for continuous, toxicity grades endpoints and regard the conventional binary endpoint as a special case.

**Result:**

The proposed gBOINS design enjoys convergence properties, e.g., the induced interval shrinks to the toxicity target and the recommended dose converges to the true maximum tolerated dose with increased sample size.

**Conclusion:**

The proposed gBOINS design is transparent and simple to implement. We show that the gBOINS design has the desirable finite property of coherence and large-sample property of consistency. Numerical studies show that the proposed gBOINS design yields good performance and is comparable with or superior to the competing design.

## Introduction

In oncology phase I trial studies, one main objective is to determine the maximum tolerated dose (MTD) or the recommended phase II dose (RP2D). Targeting on pahse I trial studies, numerous methods have been proposed and can be generally classified into three classes: *algorithm-based design* like the 3+3 design [1], the accelerated titration design [2], and the biased coin design [3]; *model-based design* like the continual reassessment method (CRM) [4, 5] and its various extensions [6–8]; and recently developed *model-assisted design* like the Bayesian optimal interval (BOIN) design [9], and the Keyboard design [10]. Note that, all these methods accounting for the binary toxicity outcomes, experienced dose-limiting toxicity (DLT) or not, and thus may not suitable for the trials with multiple low- or moderate-grade toxicities, such as the molecularly targeted or immunotherapy trials [11–14]. To incorporate the toxicity grades (please refer National Cancer Institute Common Terminology Criteria) into dose-finding trials, one way is assigning severity weights to each grade and type of toxicity and combine the weights as a composite score, eg., total toxicity burden (TTB) [15], toxicity burden score (TBS) [16], and total toxicity profile (TTP) [17]. After appropriate transformations, these scores can be taken as the normally distributed variable [14]. The other way is translating toxicity grades to numeric scores which represent their relative severity in the unit of DLT, the ‘equivalent toxicity score’ (ETS) [18] and treating them as quasi-binary end points which take the values ranging from 0 to 1 and can be modelled by the quasi-Bernoulli likelihood [19].

To the best of our knowledge, very few methods in phase I were developed to account for different toxicity scores, e.g., binary, continuous, count, in a unified framework except a design by [20] and a gBOIN design by [14]. The design by [20] is an algorithm-based design and the gBOIN is a model-assisted design, which is a generalized version of the BOIN design by [9] to account for various toxicity grades. This paper will take further steps to extend the gBOIN. The gBOIN design assumes two fixed boundaries *ϕ*_1_ and *ϕ*_2_, by which the dose transition was conducted. Though gBOIN with fixed boundaries enjoys the desirable performance in finite sample size in the previous study by [14], it leaves the potential for us to improve the performance of gBOIN by exploring its behaviors with non-fixed boundaries. The rationale of studying the non-fixed boundaries is straightforward, since different boundaries are associated with different risks of making wrong dose allocations. In this article, we propose a gBOINS design method, which generalized the gBOIN method with two shrinkage boundaries $\phi _{1}^{*}$ and $\phi _{2}^{*}$. These two boundaries are obtained based on the theory of the uniformly most powerful Bayesian test [21]. The trial will be guided by replacing the two fixed boundaries *ϕ*_1_ and *ϕ*_2_ in the gBOIN with $\phi _{1}^{*}$ and $\phi _{2}^{*}$, respectively. We show that, in contrast to the gBOIN design which will oscillate among the doses within the equivalent interval, the new proposed gBOINS design has the ideal large-sample behavior that converges to one of the dose levels within the equivalent interval, because its decision boundaries shrink to a point mass toward the target. This distinctive feature of gBOINS provides a theoretical foundation and guarantees the MTD convergence. Numerical studies also show that: for small sample size, the gBOINS yields good performance that is comparable or superior to its raw version gBOIN; for large sample size, compared to the gBOIN design, the performance of gBOINS has a substantial improvement.

Remainder of the paper is organized as follows. In “Method” section, after a brief introduction of gBOIN design we introduce the gBOINS design, its theoretical foundation and derive its properties. In “Simulation” section, we compare the gBOINS design to the gBOIN design with various types of toxicity grades. In “Conclusion” section, we conclude the paper with a discussion.

## Method

### Introduction of gBOIN design

Assume there are *J* specified doses *d*_1_<⋯<*d*_*J*_ under investigation. Let *y* denote the toxicity outcome which is either binary or quasi-binary (e.g., DLT or ETS) or continuous (e.g., TTB, TBS or TTP). For the motivating trial, after an appropriate transformation, we take the AUC as a continuous end point and model it by a normal distribution. [14] adopted the binomial and the normal distributions for binary (or quasi-binary) and continuous endpoints, respectively. Define *μ*=E(*y*) and *μ*_*j*_=E(*y*|*d*_*j*_). Given the dose *d*_*j*_, the distribution of *y* belongs to the exponential family,

1 $$ f(y|d_{j}) = h(y)\exp\left\{\eta(\theta_{j})T(y)-A(\theta_{j})\right\},  $$

where,

- *θ*_*j*_=*μ*_*j*_, *η*(*θ*_*j*_)= log{*μ*_*j*_/(1−*μ*_*j*_)}, *A*(*θ*_*j*_)=− log(1−*μ*_*j*_), *T*(*y*)=*y*, and *h*(*y*)=1, if *y* follows a binomial distribution;
- *θ*_*j*_=(*μ*_*j*_,*σ*^2^), *η*(*θ*_*j*_)=*μ*_*j*_/*σ*^2^, $A(\theta _{j}) = \mu _{j}^{2}/(2\sigma ^{2})$, *T*(*y*)=*y*, and $h(y) = \frac {1}{\sqrt {\pi }\sigma }\exp \left \{-y^{2}/(2\sigma ^{2})\right \}$, if *y* follows a normal distribution.

Let *ϕ*_0_ denote the target value of *μ* for dose finding. Specifically, for binary or quasi-binary toxicity endpoints, *ϕ*_0_ is the target DLT probability; for continuous endpoints, *ϕ*_0_ is the targeted value of the TTB, TBS or TTP. Assume there are *n*_*j*_ patients treated at dose level *d*_*j*_ and let ${\mathcal {D}}_{j} = (y_{1}, \cdots, y_{n_{j}})$ denote the observed toxicity data. Based on ${\mathcal {D}}_{j}$, the sample mean can be obtained as $\hat {\mu }_{j} = \sum _{i=1}^{n_{j}} y_{i} /n_{j}$. For the interval-based design, dose transition decisions are made by comparing $\hat {\mu }_{j}$ with the decision boundaries, *λ*_*e*_(*d*_*j*_,*n*_*j*_,*ϕ*_0_) and *λ*_*d*_(*d*_*j*_,*n*_*j*_,*ϕ*_0_). Specifically, if $\hat {\mu }_{j} < \lambda _{e}(d_{j}, n_{j}, \phi _{0})$, escalate to the higher dose level *j*+1, and if $\hat {\mu }_{j} > \lambda _{d}(d_{j}, n_{j}, \phi _{0})$, de-escalate to the lower dose level *j*−1, otherwise retain the same dose level *j*. The selection of the decision boundaries *λ*_*e*_(*d*_*j*_,*n*_*j*_,*ϕ*_0_) and *λ*_*d*_(*d*_*j*_,*n*_*j*_,*ϕ*_0_) is critical because these two parameters essentially determine operating characteristics of a design. Let the decisions retainment, escalation and de-escalation (each based on the current dose level), denoted as ${\mathcal {R}}$, ${\mathcal {E}}$ and ${\mathcal {D}}$, respectively and let $\overline {\mathcal {R}}$ denote the decisions that are complementary to ${\mathcal {R}}$ (i.e., $\overline {\mathcal {R}}$ includes ${\mathcal {E}}$ and ${\mathcal {D}}$), and $\overline {\mathcal {E}}$ and $\overline {\mathcal {D}}$ denote the decisions that are complementary to ${\mathcal {E}}$ and ${\mathcal {D}}$, respectively. Following the same rule of [9], to obtain optimal decision boundaries under some criteria, the gBOIN [14] considers three point hypotheses *H*_0_:*μ*_*j*_=*ϕ*_0_, *H*_1_:*μ*_*j*_=*ϕ*_1_, *H*_2_:*μ*_*j*_=*ϕ*_2_ and minimize an incorrect decision probability *α*,

2 $$ {}\alpha = \mathrm{P}(H_{0}) \mathrm{P}(\overline{\mathcal{R}} |H_{0}) + {\mathrm{P}}\left(H_{1}\right){\mathrm{P}}\left({\overline{\mathcal{E}} \left| {{H_{1}}} \right.} \right) + {\mathrm{P}}\left(H_{2}\right){\mathrm{P}}\left(\overline {\mathcal{D}}|H_{2} \right),  $$

where *ϕ*_1_ is a value deemed subtherapeutic such that dose escalation is warranted, and *ϕ*_2_ is a value deemed overly toxic such that dose de-escalation is required. Note that, *H*_0_ indicates that the current dose is the MTD and we should retain the current dose for the next cohort of patient; *H*_1_ indicates that the current dose is below the MTD and we should escalate the dose; and *H*_2_ indicates that the current dose is overly toxic and we should deescalate the dose. Thus, the correct decisions under hypotheses *H*_0_, *H*_1_ and *H*_2_ are retainment, escalation and de-escalation. Correspondingly, the incorrect decisions under *H*_0_, *H*_1_ and *H*_2_ are $\overline {\mathcal {R}}$, $\overline {\mathcal {E}}$ and $\overline {\mathcal {D}}$, respectively. For example, under *H*_0_ (i.e., the current dose is the target), the correct decision is to retain the current dose (i.e., ${\mathcal {R}}$), and incorrect decisions are dose escalation and de-escalation (i.e., ${\mathcal {E}}$ and ${\mathcal {D}}$). Taking a noninformative prior, i.e., P(*H*_0_)=P(*H*_1_)=P(*H*_2_)=1/3, and minimizing the incorrect decision probability *α* in Eq. (2), the decision boundaries can be obtained as (details can be found in [14]),

3 $$ \lambda_{e}^{*} = \frac{A(\vartheta_{1})-A(\vartheta_{0})}{\eta(\vartheta_{1}) - \eta(\vartheta_{0})},\quad \lambda_{d}^{*} = \frac{A(\vartheta_{2})-A(\vartheta_{0})}{\eta(\vartheta_{2}) - \eta(\vartheta_{0})}.  $$

Specifically, when *y* follows a Bernoulli or quasi-Bernoulli distribution, we have *𝜗*_*k*_=*ϕ*_*k*_, *A*(*𝜗*_*k*_)=− log(1−*ϕ*_*k*_), *η*(*𝜗*_*k*_)= log{*ϕ*_*k*_/(1−*ϕ*_*k*_)}. Then,

4 $$\begin{array}{*{20}l} {\lambda_{e}^{*}} = \frac{\log \frac{ 1-\phi_{1}}{1-\phi_{0}}}{\log\frac{\phi_{0}(1-\phi_{1})}{(1-\phi_{0})\phi_{1}}}, \qquad {\lambda_{d}^{*}} =\frac{\log\frac{1-\phi_{0}}{1-\phi_{2}}}{\log\frac{\phi_{2}(1-\phi_{0})}{(1-\phi_{2})\phi_{0}}}, \end{array} $$

which are exactly the same as boundaries provided by the original BOIN design [9]. When *y* follows a normal distribution, we have $\vartheta _{k}=\left (\phi _{k}, \sigma _{j}^{2}\right)$, $A\left (\vartheta _{k}\right) = \phi _{k}^{2}/\left (2\sigma _{j}^{2}\right)$, $\eta \left (\vartheta _{k}\right) = \phi _{k}/\sigma _{j}^{2}$. Then,

5 $$\begin{array}{*{20}l} {\lambda_{e}^{*}} =\frac{\phi_{0}+\phi_{1}}{2 },\qquad {\lambda_{d}^{*}} =\frac{\phi_{0}+\phi_{2}}{2 }. \end{array} $$

Based on the above decision boundaries, the gBOIN design is summarized as follows:

- Patients in the first cohort are treated at the lowest dose level or at a prespecified dose level.
- At the current dose level *j*, assign a dose to the next cohort of patients,
  if ${\hat \mu _{j}} \le {\lambda _{e}^{*}}$, escalate the dose level to *j*+1,
  if ${\hat \mu _{j}} \ge {\lambda _{d}^{*}}$, de-escalate the dose level to *j*−1, and
  otherwise, i.e., ${\lambda _{e}^{*}} < {\hat \mu _{j}} < {\lambda _{d}^{*}}$, retain the same dose level, *j*.
- This process is continued until the maximum sample size is reached or the trial is terminated because of excessive toxicities.

It is remarkable that the optimal decision boundaries $\left ({\lambda _{e}^{*}}, {\lambda _{d}^{*}}\right)$ are free of *d*_*j*_ and *n*_*j*_, which means that the same pair of boundaries are used throughout the trial no matter which dose is the current dose, nor how many patients have been treated at the current dose.

### Adaptive gBOIN design

Extensive simulation studies have shown that the gBOIN is transparent and simple to implement, and it yields good performance that is comparable or superior to more complicated model-based designs. As we described in the “Introduction” section, the un-fixed boundaries may allow a flexibility to penalize mis-allocation rate of patients at over-toxic doses. To account for un-fixed boundaries, firstly, we reformulate the above three hypotheses as follows,

$$H_{0}: \mu_{j} = \phi_{0} \quad versus \quad H_{1}: \mu_{j} = \phi_{1}, $$

and

$$H_{0}: \mu_{j} = \phi_{0} \quad versus \quad H_{2}: \mu_{j} = \phi_{2}. $$

In the Bayesian paradigm, the Bayes factor in favor of the alternative hypothesis *H*_1_ against a fixed null hypothesis *H*_0_ is defined as,

6 $$ \text{BF}_{10}(D_{j}) = \frac{\mathrm{P}(H_{1}|D_{j})/\mathrm{P}(H_{0}|D_{j})}{\mathrm{P}(H_{1})/\mathrm{P}(H_{0})},  $$

and the null hypothesis *H*_0_ is rejected if BF_10_(*D*_*j*_) exceeds a prespecified threshold *γ*_1_. Similarly, the Bayes factor in favor of the alternative hypothesis *H*_2_ against a fixed null hypothesis *H*_0_ is defined as,

7 $$ \text{BF}_{20}(D_{j}) = \frac{\mathrm{P}(H_{2}|D_{j})/\mathrm{P}(H_{0}|D_{j})}{\mathrm{P}(H_{2})/\mathrm{P}(H_{0})},  $$

and the null hypothesis *H*_0_ is rejected if BF_20_(*D*_*j*_) exceeds a prespecified threshold *γ*_2_. Note that, if we want to put more penalties on over-toxic allocation, values of *γ*_1_ and *γ*_2_ would be different and presumably *γ*_1_ should be greater than *γ*_2_ since smaller *γ*_2_ means decisions of de-escalation are easier made if over-toxicities occur. Given the prior odds P(*H*_*k*_)/P(*H*_0_)=1 and the threshold *γ*_*k*_,(*k*=1,2), we can determine an alternative hypothesis that maximize the probability that the Bayes factor forms a test exceed the specified threshold *γ*_*k*_. In other words, here we can choose the value of $\phi _{k}^{*}, (k=1,2)$ (this notation has been introduced in the “Introduction” section) to maximize P(BF_*k*0_(*D*_*j*_)>*γ*_*k*_).

By the Lemma 1 of [21], $\phi _{1}^{*}$ and $\phi _{2}^{*}$ can be obtained by,

8 $$\begin{array}{*{20}l} {}\phi_{1}^{*} = \underset{\mu_{j}<\phi_{0}}{\arg\max}~g_{\gamma_{1}}(\mu_{j}, \phi_{0}) \quad and \quad \phi_{2}^{*} = \underset{\mu_{j}>\phi_{0}}{\arg\min}~g_{\gamma_{2}}(\mu_{j}, \phi_{0}) \end{array} $$

respectively, where $g_{\gamma _{k}}(\mu _{j}, \phi _{0}) = \frac {\log (\gamma _{k})+n_{j}\{A(\theta _{j})-A(\theta _{0})\}}{\eta (\theta _{j})-\eta (\theta _{0})}$, *k*=1,2.

Specifically, for binomial distribution, $\phi _{1}^{*}$ and $\phi _{2}^{*}$ can be given as,

9 $$\begin{array}{*{20}l} {}&\phi_{1}^{*} = \underset{\mu_{j}<\phi_{0}}{\arg\max}~\frac{\log(\gamma_{1})-n_{j}\left\{\log(1-\mu_{j})-\log(1-\phi_{0})\right\}}{\log\left\{\mu_{j}/(1-\mu_{j})\right\} - \log\left\{\phi_{0}/(1-\phi_{0})\right\}}, \\ {}&\phi_{2}^{*} = \underset{\mu_{j}>\phi_{0}}{\arg\min}~\frac{\log(\gamma_{2})-n_{j}\left\{\log(1-\mu_{j})-\log(1-\phi_{0})\right\}}{\log\left\{\mu_{j}/(1-\mu_{j})\right\} - \log\left\{\phi_{0}/(1-\phi_{0})\right\}}. \end{array} $$

Obviously, the values of $\phi _{1}^{*}$ and $\phi _{2}^{*}$ depend on the target *ϕ*_0_, the sample size *n*_*j*_ and the threshold *γ*_*k*_, *k*=1,2. Although their close forms cannot be obtained, they can be solved via numerical optimization methods. For normal distribution, $\phi _{1}^{*}$ and $\phi _{2}^{*}$ can be given as,

10 $$\begin{array}{*{20}l} & \phi_{1}^{*} = \phi_{0} - \sigma\sqrt{\frac{2\log\gamma_{1}}{n_{j}}}, \\ & \phi_{2}^{*} = \phi_{0} + \sigma\sqrt{\frac{2\log\gamma_{2}}{n_{j}}}. \end{array} $$

Note that, for the normal distribution, values of $\phi _{1}^{*}$ and $\phi _{2}^{*}$ depend on the value of *σ*. So, if *σ* is unknown, we can replace it with its sample estimation $\hat {\sigma } = \sqrt {\left \{\sum _{i=1}^{n_{j}} \left (y_{i} - \hat {\mu }_{j}\right)^{2}\right \}/n_{j}}$, or alternatively, we can take an Inverse Gamma distribution with shape parameter *α*_0_ and rate parameter *β*_0_ as its prior, then *σ* can be replaced by using its posterior mean $\left (2\beta _{0} + \sum _{i=1}^{n_{j}} \left (y_{i} - {\mu }_{j}\right)^{2}\right)/ \left (n_{j} + \alpha _{0}\right)$ with *μ* replaced by $\hat {\mu }_{j}$.

Replacing *ϕ*_*k*_ in $\lambda _{e}^{*}$ and $\lambda _{d}^{*}$ with $\phi _{k}^{*}$, *k*=1,2, we can get the adaptive shrinkage decision boundaries $\lambda _{e}^{*}(n_{j})$ and $\lambda _{d}^{*}(n_{j})$. Note that, for a standard binary toxicity endpoint, if we take the same values for *γ*_*k*_, *k*=1,2, the boundaries are the same as the UMPBI design [22]. Based on Lemma 2 in [21], we have the following double-shrinkage property theorem about the shrinkage boundaries $\lambda _{e}^{*}(n_{j})$ and $\lambda _{d}^{*}(n_{j})$.

#### **Theorem 1**

As *n*_*j*_→*∞*, the decision boundaries $\lambda _{e}^{*}(n_{j})$ and $\lambda _{d}^{*}(n_{j})$ will converge to the target *ϕ*_0_ at the rate of $O\bigl (\sqrt {\log (\gamma _{1})/n_{j}}\bigr)$ and $O\bigl (\sqrt {\log (\gamma _{2})/n_{j}}\bigr)$ respectively.

Theorem 1 introduces a double-shrinkage property for the proposed adaptive gBOIN design: The optimal values $\phi _{k}^{*}$ shrink toward the target toxicity probability *ϕ*_0_, and the optimal boundaries $\lambda _{e}^{*}(n_{j})$ and $\lambda _{d}^{*}(n_{j})$ based on each combination of $\phi _{1}^{*}$ and $\phi _{2}^{*}$ shrinkage toward the target value *ϕ*_0_.

Now we give the procedure of the proposed gBOINS design as follows.

- Patients in the first cohort are treated at the lowest dose level or at a prespecified dose level.
- At the current dose level *j*, to assign a dose to the next cohort of patients,
  if ${\hat \mu _{j}} \le {\lambda _{e}^{*}(n_{j})}$, escalate the dose level to *j*+1,
  if ${\hat \mu _{j}} \ge {\lambda _{d}^{*}(n_{j})}$, de-escalate the dose level to *j*−1, and
  otherwise, i.e., ${\lambda _{e}^{*}(n_{j})} < {\hat \mu _{j}} < {\lambda _{d}^{*}(n_{j})}$, retain the same dose level, *j*.
- This process is continued until the maximum sample size is reached or the trial is terminated because of excessive toxicities.

After the trial has been completed, we use the pooled adjacent violators algorithm [23] to select a dose level as the MTD. Denote the isotonically transformed values of the observed value $\{\hat \mu _{j}\}$ by $\{\tilde \mu _{j}\}$, to be specific, for finding the MTD, we select dose *j*^∗^, for which the isotonic estimate of the toxicity rate $\tilde \mu _{j^{*}}$ is closest to *ϕ*_0_; if there are ties for $\tilde \mu _{j^{*}}$, we select from the ties the highest dose level when $\tilde \mu _{j^{*}} < \phi _{0}$ or the lowest dose level when $\tilde \mu _{j^{*}} >\phi _{0}$.

For patient safety, we impose the following overdose control rule when using the gBOIN design.

If ${\mathrm {P}\left ({{\mu _{j}} > \phi _{0} \left | {\mathcal {D}}_{j} \right.} \right) > 0.95}$ and *n*_*j*_≥3, dose levels *j* and higher are eliminated from the trial, and the trial is terminated if the first dose level is eliminated.

Posterior probability ${\mathrm {P}\left ({{\mu _{j}} > \phi _{0} \left | {\mathcal {D}}_{j} \right.} \right) > 0.95}$ can be evaluated on the basis of a beta-binomial model for the binary or quasi-binary endpoint, assuming *μ*_*j*_ follows a vague beta prior, e.g., *μ*_*j*_∼*beta*(1,1). For normal endpoint *y* with mean *μ*_*j*_ and variance $\sigma _{j}^{2}$, assuming noninformative prior $(\mu, \sigma _{j}^{2}) \propto \sigma ^{-2}$, the posterior distribution of *μ*_*j*_ follows a *t* distribution with *n*_*j*_−1 degrees of freedom, mean $\hat \mu _{j}$ and scale $n_{j}^{-1}\sum _{i=1}^{n_{j}}\left (y_{i} - \hat \mu _{j}\right)^{2}$.

### Design properties

From a practical viewpoint, a natural requirement for dose-finding trials is that dose escalation should be not allowed if the observed toxicity rate or mean toxicity score at the current dose is higher than the target, and dose de-escalation should not be allowed if the observed toxicity rate or mean toxicity score at the current dose is lower than the target. [9] referred to this finite sample property as “long-term memory coherent”, which is an extension of a similar concept originally proposed by [24]. That original definition of design coherence requires the prohibition of dose escalation (or de-escalation) when the observed toxicity rate in the most recently treated cohort is more (or less) than the target toxicity rate. Because that definition is based on the response from only the most recently treated cohort without considering responses from patients who were previously enrolled and treated, [9] refers this definition as “short-term memory coherence”. Clearly, short-term memory coherence is a stronger counterpart than long-term memory coherence.

As shown in the Appendix, the gBOINS design has the following desirable finite-sample property.

#### **Theorem 2**

The gBOINS design is long-term memory coherent in the sense that the design will never escalate the dose when $\hat {\mu }_{j}>\phi _{0}$; and will never de-escalate the dose when $\hat {\mu }_{j}<\phi _{0}$.

To further enhance safety of the design, we let the upper boundary $\phi _{2}^{*}$ have a little bit faster shrinking rate than that of the lower boundary $\phi _{1}^{*}$, since more strict or smaller $\phi _{2}^{*}$ has less risk of exposing participated patients to over-toxic doses. We propose to take *γ*_*k*_ as $\gamma _{k} = \exp (c_{k}n_{j}^{\varepsilon _{k}})$, *k*=1,2, 1>*ε*_1_≥*ε*_2_>0 and $0< c_{1} < n_{j}^{1-\epsilon _{1}}\log (1/(1-\phi _{0}))$ and $c_{1}< c_{2} < n_{j}^{1-\epsilon _{2}}\log (1/\phi _{0})$. It can be shown that the proposed adaptive gBOIN design has the following desirable large-sample property.

#### **Theorem 3**

As the number of patients goes to infinity, the dose assignment and the selection of the MTD under the gBOINS design converge almost surely to dose level *j*^∗^, if $\phantom {\dot {i}\!}\mu _{j^{*}} = \phi _{0}$.

According to Theorem 1, the condition $\gamma _{k} = \exp (c_{k}n_{j}^{\varepsilon _{k}})$, *c*_*k*_>0, *k*=1,2, imposed here to leverage the converge rate of $\lambda _{e}^{*}{(n_{j})}$ and $\lambda _{d}^{*}{(n_{j})}$, yielding $\mathrm {P}\{\hat {\mu }_{j} \in (\lambda _{e}^{*}{(n_{j})}, \lambda _{d}^{*}{(n_{j})})\} =1 $, because $\hat {\mu }_{j}$ converges in probability to *μ*_*j*_ at the $\sqrt {n}$ rate. Following the proof of Theorem 1 of [25], the result can be directly obtained and is omitted here.

### Practical implementation

To implement the proposed gBOINS design in practice, we need to specify the values of *ε*_*k*_ and *c*_*k*_, *k*=1,2. We recommend the *ε*_*k*_=0.5, *k*=1,2. The values of *c*_*k*_, *k*=1,2 need to be calibrated by extensive simulation studies, and even there are no uniform values for different type of endpoints with the same target. For the normal endpoints, the shrinkage boundaries depend on the estimate of *σ*, this will influence the pre-tabulation and the simplicity of gBOINS. For practical applications, we suggest to replace it with 1.1*ϕ*_0_. Note that a big (small) value of $\lambda _{e}^{*}(n_{j})$ (or $\lambda _{d}^{*}(n_{j})$) will make dose escalation (de-escalation) rapidly, this may lead serious safety problems and reduce the efficiency of the design when the sample size is small, since the smaller value of the sample size the bigger variance of $\hat {\mu }_{j}$. To avoid this adverse event problem and improve the design’s efficiency, in practice, we introduce a lead-in process in a trial to follow the original gBOIN design for a pre-specified number of patients (denoted as *N*_0_). After *n*_*j*_>*N*_0_, the trial is then switched to the gBOINS design. For our simulations, *N*_0_=6 is recommended. Table 1 shows examples of the values of $(\lambda _{e}^{*}(n_{j}), \lambda _{d}^{*}(n_{j}))$ for target *ϕ*_0_=0.2 and *ϕ*_0_=0.3.

**Table 1:** Dose escalation and de-escalation boundaries for Bernoulli and continuous toxicity endpoint, with *ϕ*_0_=0.2, *ϕ*_0_=0.3, *ε*_*k*_=0.5, *k*=1,2 and *N*_0_=6

End point		*n*_*j*_	3	6	9	12	15	18	21	24	27	30
Bernoulli	*ϕ*_0_=0.2	$\lambda _{e}^{*}{(n_{j})}$	0.16	0.16	0.16	0.17	0.17	0.17	0.17	0.17	0.17	0.17
or		$\lambda _{d}^{*}{(n_{j})}$	0.24	0.24	0.22	0.22	0.22	0.22	0.22	0.22	0.22	0.22
quasi-	*ϕ*_0_=0.3	$\lambda _{e}^{*}{(n_{j})}$	0.24	0.24	0.24	0.25	0.25	0.25	0.25	0.25	0.26	0.26
Bernoulli		$\lambda _{d}^{*}{(n_{j})}$	0.36	0.36	0.33	0.33	0.33	0.33	0.33	0.33	0.33	0.32
Continuous	*ϕ*_0_=0.2	$\lambda _{e}^{*}{(n_{j})}$	0.16	0.16	0.17	0.17	0.18	0.18	0.18	0.18	0.18	0.18
		$\lambda _{d}^{*}{(n_{j})}$	0.24	0.24	0.22	0.21	0.21	0.21	0.21	0.21	0.21	0.21
	*ϕ*_0_=0.3	$\lambda _{e}^{*}{(n_{j})}$	0.24	0.24	0.26	0.26	0.27	0.27	0.27	0.27	0.27	0.27
		$\lambda _{d}^{*}{(n_{j})}$	0.36	0.36	0.32	0.32	0.32	0.32	0.32	0.32	0.32	0.32

For the binary endpoint, *c*_1_= log(1.05) and *c*_2_= log(1.05)/3 for *ϕ*_0_=0.2, and *c*_1_= log(1.1) and *c*_2_= log(1.1)/3 for *ϕ*_0_=0.3. For the continuous end point, *c*_1_= log(1.1) and *c*_2_= log(1.1)/3 for both *ϕ*_0_=0.2 and *ϕ*_0_=0.3

## Simulation

### Toxicity as a binary endpoint

We test the performance of the gBOINS design by comparing it to the gBOIN design under four different metrics: the percentage of correct selection (PCS) of the MTD, the average number of patients allocated to the MTD, the risk of overdosing, which is defined as the percentage of simulated trials in which a large percentage (e.g., more than 60% or 80%) of patients are treated at doses above the MTD and the risk of underdosing which is defined as the percentage of simulated trials in which more than 80% of patients are treated at doses below the MTD. We investigated two target toxicity rates *ϕ*_0_=0.2 and *ϕ*_0_=0.3, and for each of the target toxicity rate, we examined 16 representative toxicity scenarios with various parameters of *ϕ*_0_=0.2, *c*_1_ = log(1.05)/3 and *c*_2_ = log(1.05)/3, and *c*_1_ = log(1.1)/3 and *c*_2_ = log(1.1)/3 when *ϕ*_0_=0.3. Table 2 which were reproduced from [27]. All examined scenarios are varied in the location of the MTD and the gaps around the MTD. For each scenario, 30 patients and 10 cohorts were assumed.

**Table 2:** Sixteen true toxicity scenarios reproduced from [26], with the target DLT rates of 0.2 and 0.3 in boldface

	Dose Level							Dose Level				
Scenario	1	2	3	4	5		Scenario	1	2	3	4	5
1	**0.20**	0.25	0.35	0.45	0.50		1	**0.30**	0.40	0.50	0.60	0.70
2	**0.20**	0.30	0.40	0.50	0.60		2	**0.30**	0.45	0.60	0.70	0.80
3	0.15	**0.20**	0.25	0.35	0.45		3	0.20	**0.30**	0.40	0.50	0.60
4	0.15	**0.20**	0.30	0.45	0.55		4	0.20	**0.30**	0.45	0.60	0.70
5	0.10	**0.20**	0.25	0.35	0.45		5	0.15	**0.30**	0.40	0.50	0.60
6	0.10	**0.20**	0.30	0.40	0.55		6	0.15	**0.30**	0.45	0.60	0.70
7	0.08	0.15	**0.20**	0.25	0.35		7	0.12	0.20	**0.30**	0.40	0.50
8	0.08	0.15	**0.20**	0.30	0.45		8	0.12	0.20	**0.30**	0.45	0.60
9	0.05	0.10	**0.20**	0.25	0.35		9	0.05	0.15	**0.30**	0.40	0.50
10	0.05	0.10	**0.20**	0.30	0.45		10	0.05	0.15	**0.30**	0.45	0.60
11	0.05	0.10	0.15	**0.20**	0.25		11	0.05	0.12	0.20	**0.30**	0.40
12	0.05	0.10	0.15	**0.20**	0.30		12	0.05	0.12	0.20	**0.30**	0.45
13	0.02	0.06	0.10	**0.20**	0.25		13	0.02	0.08	0.15	**0.30**	0.40
14	0.02	0.06	0.10	**0.20**	0.30		14	0.02	0.08	0.15	**0.30**	0.45
15	0.02	0.05	0.07	0.10	**0.20**		15	0.02	0.10	0.15	0.20	**0.30**
16	0.01	0.06	0.10	0.15	**0.20**		16	0.01	0.04	0.08	0.15	**0.30**

**Fig. 1:**
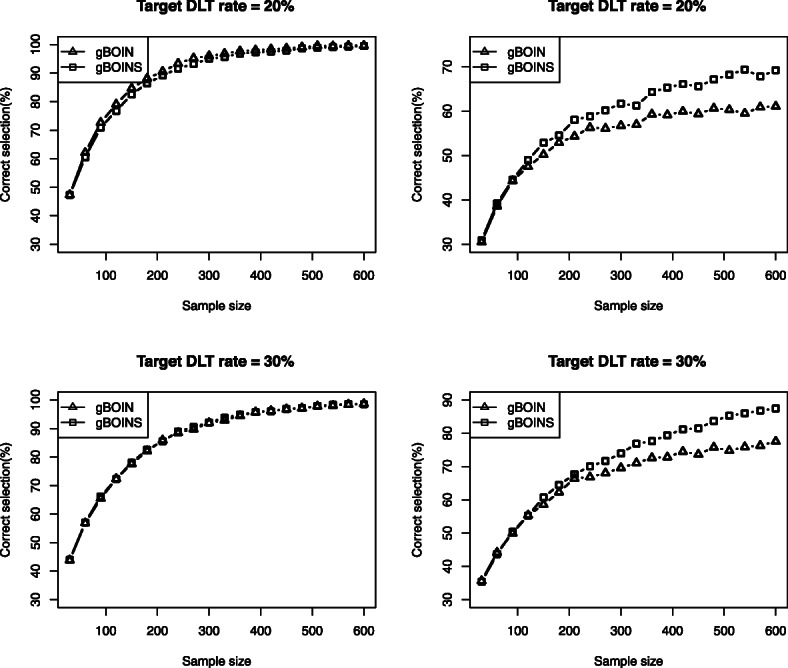
The operating characteristics of gBOIN and gBOINS when the target toxicity rate is 20%

**Fig. 2:**
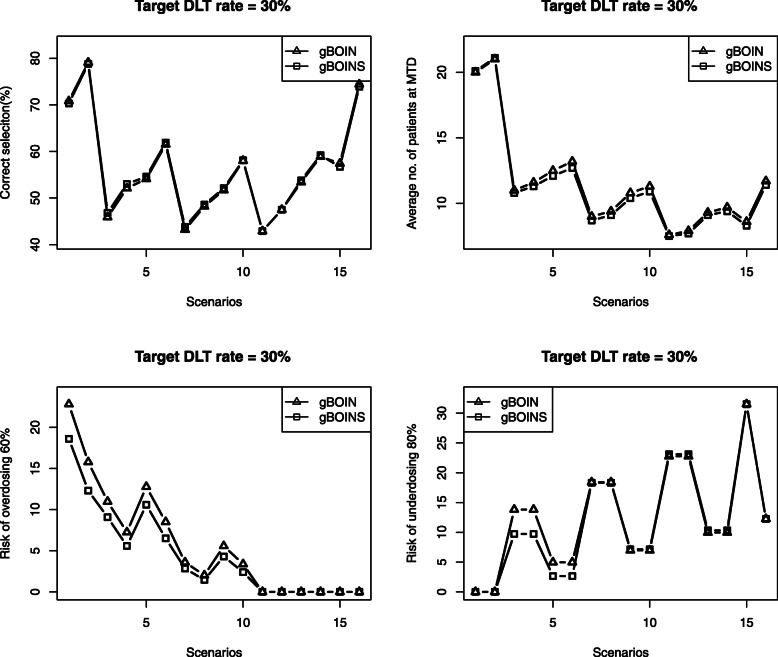
The operating characteristics of gBOIN and gBOINS when the target toxicity rate is 30%

Figures 1 and 2 present the results based on 4000 simulated trials. As shown in Fig. 1, when the target is 0.2, for all 16 scenarios the performance of gBOINS and gBOIN are comparable in the sense of percentage of correct selection of the target dose and the average number of patients allocated to the MTD. While the gBOIN has a higher risk of overdosing, for most scenarios, acromm scenarios 1 to 10. In addition, compared to the gBOIN design, the proposed gBOINS allocated fewer patients to sub-therapeutic doses for most scenarios, which may be explained by a higher risk of underdosing 80% for the gBOIN. Figure 2 shows that, when the target is 0.3, the performance of gBOINS and gBOIN are comparable, and the proposed gBOINS has a lower risk of overdosing.

**Fig. 3:**
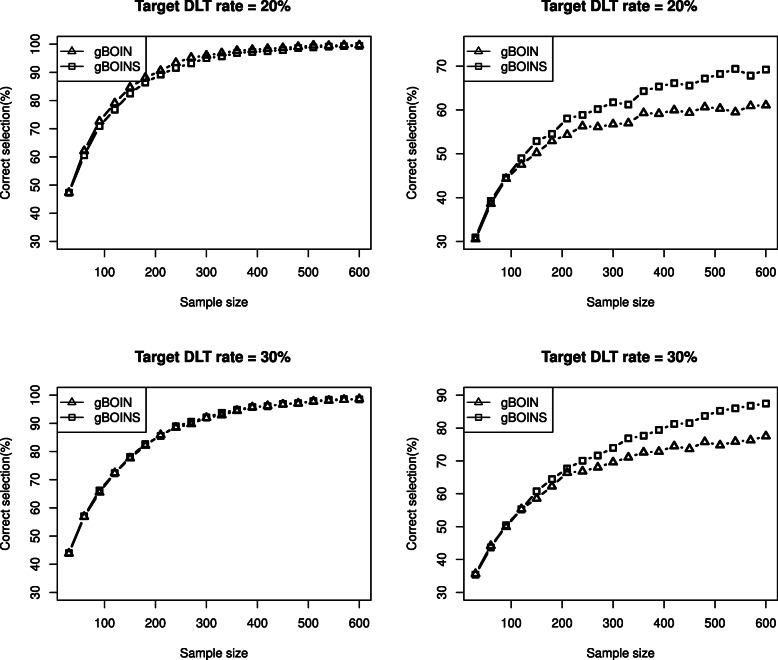
The relationships of operating characteristics and sample sizes of gBOIN and gBOINS when the target toxicity rates are 20% and 30%. The two pictures on the left panel, only one dose lying inside (0.16,0.24) and (0.24,0.36) respectively for the top and bottom panel; on the right panel, there are two doses lying inside their corresponding intervals

At the end of the “Toxicity as a binary endpoint” section, we also conducted simulation studies to investigate the performance of the gBOINS with respect to different sample sizes. We consider four scenarios in Table 3 and the simulation results based on 4000 replications are presented in Fig. 3. As shown in the first two pictures on the left panel of Fig. 3, for scenarios 1 and 3, there was only one dose lying inside the interval (0.16,0.24) and (0.24,0.36) respectively, the performance of gBOINS is comparable to the gBOIN design. For scenarios 2 and 4, the simulation results are depicted on the right panel of Fig. 3, there were two doses lying inside the intervals and the gBOINS outperformed the gBOIN when the sample size was greater than 90.

**Table 3:** Four true toxicity scenarios with the target DLT rates of 0.2 and 0.3 in boldface

	Target DLT rate =20*%*						Target DLT rate =30*%*				
Scenario	1	2	3	4	5	Scenario	1	2	3	4	5
1	0.01	0.10	0.20	0.30	0.35	3	0.01	0.20	0.30	0.40	0.50
2	0.01	0.18	0.20	0.30	0.35	4	0.01	0.25	0.30	0.40	0.50

### Toxicity as a quasi-binary endpoint

We evaluated the performance of the gBOINS design when the toxicity endpoint was a quasi-binary endpoint (e.g., ETS) by comparing it to the gBOIN design method based on the ten scenarios considered by [14](see Table 4). Following [18], we adopted the following ETS definition: grades 0 and 1 were of no concern (no DLT); a grade-2 toxicity was equivalent to 0.5 DLT; a grade-3 toxicity counted as one DLT; and a grade-4 toxicity was equivalent to 1.5 DLT. The target ETS was 0.47, derived from the target profile of 49% grade 0 and grade 1, 18% grade 2, 23% grade 3, and 10% grade 4. That is, the target ETS = 0.49×0+0.18×0.5+0.23×1.0+0.10×1.5=0.47. A total sample of 30 patients in 10 cohorts was used in the simulation, with *d*_1_ as the starting dose. And the *c*_*k*_, *k*=1,2, are set to be *c*_1_ = log(1.2)/3 and *c*_2_ = log(1.2) throughout this subsection.

**Table 4:** True probability of each toxicity grade (0/1, 2, 3, 4) at each dose level (1-6) for ten simulation scenarios (1-10) [18]

Grade	*d*_1_	*d*_2_	*d*_3_	*d*_4_	*d*_5_	*d*_6_		*d*_1_	*d*_2_	*d*_3_	*d*_4_	*d*_5_	*d*_6_
	Scenario 1							Scenario 2					
0,1	0.83	0.75	0.62	0.51	0.34	0.19		0.92	0.85	0.70	0.55	0.24	0.00
2	0.12	0.15	0.18	0.19	0.16	0.11		0.03	0.05	0.10	0.15	0.26	0.36
3	0.04	0.07	0.11	0.14	0.15	0.11		0.03	0.07	0.14	0.21	0.35	0.49
4	0.01	0.03	0.09	0.16	0.35	0.59		0.02	0.03	0.06	0.09	0.15	0.21
	Scenario 3							Scenario 4					
0,1	0.78	0.56	0.50	0.40	0.30	0.16		0.88	0.64	0.52	0.35	0.17	0.00
2	0.14	0.19	0.18	0.17	0.15	0.09		0.04	0.12	0.16	0.22	0.28	0.39
3	0.06	0.12	0.14	0.15	0.14	0.10		0.06	0.17	0.22	0.30	0.38	0.52
4	0.02	0.12	0.18	0.28	0.41	0.65		0.02	0.07	0.10	0.13	0.17	0.23
	Scenario 5							Scenario 6					
0,1	1.00	0.91	0.88	0.86	0.80	0.65		0.50	0.38	0.29	0.19	0.13	0.08
2	0.00	0.06	0.07	0.08	0.10	0.13		0.25	0.24	0.21	0.16	0.11	0.07
3	0.00	0.03	0.04	0.05	0.08	0.14		0.11	0.12	0.12	0.10	0.08	0.05
4	0.00	0.00	0.01	0.01	0.02	0.08		0.14	0.26	0.38	0.55	0.68	0.80
	Scenario 7							Scenario 8					
0,1	0.78	0.58	0.50	0.40	0.30	0.16		0.92	0.76	0.68	0.57	0.45	0.25
2	0.14	0.18	0.18	0.17	0.15	0.09		0.00	0.00	0.00	0.00	0.00	0.00
3	0.00	0.00	0.00	0.00	0.00	0.00		0.08	0.24	0.32	0.43	0.55	0.75
4	0.08	0.24	0.32	0.43	0.55	0.75		0.00	0.00	0.00	0.00	0.00	0.00
	Scenario 9							Scenario 10					
0,1	0.66	0.10	0.00	0.00	0.00	0.00		0.84	0.52	0.36	0.14	0.45	0.25
2	0.34	0.90	0.86	0.54	0.20	0.33		0.16	0.48	0.64	0.86	0.00	0.00
3	0.00	0.00	0.14	0.46	0.80	0.00		0.00	0.00	0.00	0.00	0.55	0.75
4	0.00	0.00	0.00	0.00	0.00	0.67		0.00	0.00	0.00	0.00	0.00	0.00

Table 5 shows the results based on 4,000 simulated trials. In general, for Scenarios 1, 2, 3, 4, 5 and 8, the performance of gBOINS are comparable with the gBOIN design in terms of PCS, number of patients allocating to the MTD, while the gBOIN assigns more patients to the overly toxic doses above the MTD. For example, in scenario 3, in which dose level 2 was the MTD, the gBOINS design yielded a PCS of 48% and allocated 14.5 patients to the MTD; the gBOIN yielded a PCS of 47% and allocated 12.1 patients to the MTD. While the gBOINS assigned 2.7 fewer patients than the gBOIN design to the overly toxic doses. In scenario 6, in which the MTD was dose level 1, the PCS of the gBOINS was 77% and has a 8% higher than that of the gBOIN design. In scenario 7, the MTD was located at the lose level 2 and the gBOIN design yielded a of 55%, which was 4% higher than that of the gBOINS. While the gBOINS allocated more patients to the MTD and assigned fewer patients to the overly toxic doses. In scenario 9, in which dose level 2 was the MTD, the gBOINS design yielded a PCS of 96% and was 5% higher than that of the gBOIN design. The number of patients allocated to MTD was similar, while the gBOINS assigned 3.4 fewer patients than the gBOIN design to the overly toxic doses. In scenario 10, in which dose level 4 was the MTD, the gBOINS design yielded a PCS of 76% and was 5% higher than that of the gBOIN design. The gBOIN design assigned 13.3 patients to MTD and was higher than that of the gBOINS, while gBOINS assigned fewer patients to the overly toxic doses. In addition, the gBOINS design yielded a 6% chose the overly toxic doses as MTD and had a substantially improvement compared with the gBOIN design which has a 26% chose the overly toxic doses as the MTD.

**Table 5:** Simulation results comparing the gBOINS design with the gBOIN when the equivalent toxicity score (ETS) is used as the quasi-binary toxicity endpoint. The target dose (i.e., MTD) is in boldface

		Selection percentage							Average number of patients treated					
		*d*_1_	*d*_2_	*d*_3_	*d*_4_	*d*_5_	*d*_6_		*d*_1_	*d*_2_	*d*_3_	*d*_4_	*d*_5_	*d*_6_
Scenario 1														
ETS		0.12	0.19	0.34	**0.48**	0.76	1.05							
gBOIN		0.00	0.02	0.29	**0.56**	0.12	0.01		3.5	5.1	8.4	**8.9**	3.7	0.4
gBOINS		0.00	0.03	0.32	0.53	0.12	0.00		4.7	6.8	10.2	**7.1**	1.3	0.0
Scenario 2														
ETS		0.08	0.14	0.28	**0.42**	0.70	0.98							
gBOIN		0.00	0.00	0.16	**0.66**	0.18	0.00		3.5	4.4	7.5	**9.9**	4.3	0.4
gBOINS		0.00	0.01	0.14	0.63	0.22	0.00		3.8	5.0	8.7	9.5	3.0	0.1
Scenario 3														
ETS		0.16	**0.40**	0.50	0.66	0.83	0.12							
gBOIN		0.02	**0.47**	0.36	0.13	0.00	0.00		6.3	**12.1**	7.7	3.2	0.5	0.0
gBOINS		0.06	0.48	0.36	0.09	0.01	0.00		6.8	14.5	7.0	1.6	0.1	0.0
Scenario 4														
ETS		0.11	0.34	**0.45**	0.60	0.78	1.06							
gBOIN		0.01	0.28	**0.47**	0.23	0.01	0.00		4.8	9.0	**10.8**	4.4	0.9	0.1
gBOINS		0.01	0.27	0.50	0.21	0.01	0.00		5.0	11.8	10.0	3.0	0.2	0.0
Scenario 5														
ETS		0.00	0.06	0.09	0.10	0.16	**0.32**							
gBOIN		0.00	0.00	0.00	0.00	0.04	**0.96**		3.0	3.1	3.3	3.4	4.6	**12.6**
gBOINS		0.00	0.00	0.00	0.00	0.06	0.94		3.0	3.8	4.2	4.3	5.2	9.4
Scenario 6														
ETS		**0.44**	0.63	0.80	1.01	1.16	1.29							
gBOIN		**0.69**	0.29	0.01	0.00	0.00	0.00		**19.4**	8.0	1.7	0.2	0.0	0.0
gBOINS		0.77	0.21	0.02	0.00	0.00	0.00		22.9	6.5	0.7	0.0	0.0	0.0
Scenario 7														
ETS		0.19	**0.45**	0.57	0.73	0.9	1.17							
gBOIN		0.11	**0.55**	0.28	0.06	0.00	0.00		8.9	**13.0**	6.2	1.7	0.2	0.0
gBOINS		0.15	0.51	0.30	0.04	0.00	0.00		8.8	14.6	5.6	0.9	0.0	0.0
Scenario 8														
ETS		0.08	0.24	0.32	**0.43**	0.55	0.75							
gBOIN		0.00	0.06	0.22	**0.41**	0.28	0.04		2.7	5.6	7.2	**8.5**	4.4	1.6
gBOINS		0.00	0.05	0.28	0.42	0.23	0.03		3.9	7.8	9.3	6.7	2.1	0.2
Scenario 9														
ETS		0.19	**0.45**	0.57	0.73	0.9	1.17							
gBOIN		0.00	**0.91**	0.09	0.00	0.00	0.00		3.2	**22.7**	4.1	0.0	0.0	0.0
gBOINS		0.00	0.96	0.04	0.00	0.00	0.00		5.6	23.7	0.7	0.0	0.0	0.0
Scenario 10														
ETS		0.08	0.24	0.32	**0.43**	0.55	0.75							
gBOIN		0.00	0.00	0.03	**0.71**	0.23	0.03		3.0	3.5	5.0	**13.3**	3.8	1.3
gBOINS		0.00	0.00	0.19	0.76	0.05	0.01		4.3	7.0	10.4	8.1	0.2	0.0

### Continuous outcomes

In this section, we consider ten scenarios with continuous outcomes in Table 6, all responses follow the normal distribution adopted by [20]. For the first six scenarios, the response *y* at the dose level *x*∈{1,2,3,4,5,6} follows a normal distribution *N*(0.05+0.05*x*,0.05^2^*x*^2^) and when the target at *x*=1, a sample size of 15 was used and when the target at other dose levels, a sample size of 60 was used. Cohort size of 1 was used for all scenarios. For the rest scenarios, scenarios 7 to 10, the response *y* at the dose *x*∈{1,2,3,4,5,6} also followed the normal distribution *N*(0.05+0.05*x*,0.05^2^*x*^2^), and a moderate large sample size of 100 was used. And the *c*_*k*_, *k*=1,2, are set to be *c*_1_ = log(1.1)/3 and *c*_2_ = log(1.1) throughout this subsection.

**Table 6:** Simulation results comparing gBOINS with gBOIN, when continuous toxicity end point is used. The target, correct selection percentage and correct allocation number for each scenario are in boldface

		Selection percentage							Allocation					
		*d*_1_	*d*_2_	*d*_3_	*d*_4_	*d*_5_	*d*_6_		*d*_1_	*d*_2_	*d*_3_	*d*_4_	*d*_5_	*d*_6_
Scenario 1		**0.10**	0.15	0.20	0.25	0.30	0.35							
gBOIN		**0.93**	0.06	0.01	0.00	0.00	0.00		**11.9**	2.4	0.5	0.1	0.0	0.0
gBOINS		0.93	0.06	0.00	0.00	0.00	0.00		12.0	2.4	0.5	0.1	0.0	0.0
Scenario 2		0.10	**0.15**	0.20	0.25	0.30	0.35							
gBOIN		0.03	**0.91**	0.06	0.00	0.00	0.00		7.4	**42.6**	8.2	1.4	0.3	0.1
gBOINS		0.02	0.92	0.06	0.00	0.00	0.00		11.6	37.5	8.8	1.6	0.4	0.1
Scenario 3		0.10	0.15	**0.20**	0.25	0.30	0.35							
gBOIN		0.00	0.12	**0.76**	0.12	0.01	0.00		1.6	13.5	**32.3**	9.9	2.2	0.5
gBOINS		0.00	0.09	0.78	0.12	0.01	0.00		1.6	15.4	30.2	9.7	2.4	0.6
Scenario 4		0.10	0.15	0.20	**0.25**	0.30	0.35							
gBOIN		0.00	0.00	0.23	0.60	0.16	0.02		1.1	3.0	17.5	25.6	9.8	3.0
gBOINS		0.00	0.00	0.20	0.62	0.16	0.02		1.1	3.3	18.6	24.3	9.6	3.1
Scenario 5		0.10	0.15	0.20	0.25	**0.30**	0.35							
gBOIN		0.00	0.00	0.00	0.30	0.50	0.19		1.0	1.6	5.0	19.5	21.1	11.9
gBOINS		0.00	0.00	0.00	0.28	0.53	0.19		1.0	1.6	5.6	19.4	21.0	11.4
Scenario 6		0.10	0.15	0.20	0.25	0.30	**0.35**							
gBOIN		0.00	0.00	0.00	0.03	0.35	0.61		1.0	1.2	2.4	7.7	19.6	28.0
g BOINS		0.00	0.00	0.00	0.02	0.34	0.64		1.0	1.2	2.4	7.7	20.1	27.6
Scenario 7		0.10	0.15	**0.20**	0.25	0.30	0.35							
gBOIN		0.00	0.06	0.84	0.10	0.00	0.00		1.6	16.6	64.0	14.7	2.6	0.6
gBOINS		0.00	0.04	0.89	0.07	0.00	0.00		1.6	20.3	61.7	14.5	1.6	0.3
Scenario 8		0.10	0.15	0.20	**0.25**	0.30	0.35							
gBOIN		0.00	0.00	0.17	0.69	0.13	0.01		1.1	2.9	25.2	50.9	16.1	3.8
gBOINS		0.00	0.00	0.12	0.75	0.13	0.01		1.1	3.2	26.7	51.2	15.6	3.1
Scenario 9		0.10	0.15	0.20	0.25	**0.30**	0.35							
gBOIN		0.00	0.00	0.00	0.26	0.57	0.18		1.0	1.6	5.0	30.1	42.6	19.7
gBOINS		0.00	0.00	0.00	0.20	0.66	0.14		1.0	1.6	5.4	30.4	43.4	18.1
Scenario 10		0.10	0.15	0.20	0.25	0.30	**0.35**							
gBOIN		0.00	0.00	0.00	0.01	0.33	0.66		1.0	1.2	2.5	8.6	32.7	54.1
gBOINS		0.00	0.00	0.00	0.00	0.27	0.73		1.0	1.2	2.5	8.7	34.1	52.5

Table 6 shows that when the sample size is 15 for scenario 1 and 60 for scenarios 2 to 6, performance of the gBOINS design is comparable with the gBOIN design, in correct selection percentage and number of patients treated at the target dose. While for the last four scenarios, when the sample size is moderate large, the gBOINS outperformed the gBOIN design in correct selection percentage and was comparable with gBOIN design in number of patients allocated to the target dose. Specifically, in scenario 7, in which dose level 3 was the target dose, gBOINS yielded a PCS of 89% and allocated 61.7 patients to the MTD, whereas the gBOIN yielded a PCS of 84% and allocated 64 patients to the MTD. In scenario 8, the MTD was located at the dose level 4, the PCS of gBOINS was 75% and 5% higher than that of the gBOIN. In scenarios 9-10, we also see that the PCS of gBOINS was superior to the gBOIN in PCS and was comparable with the gBOIN in the number of patients allocated to the MTD.

## Conclusion

We proposed a new phase I trial design that incorporates toxicity grades into the dose finding trials. The proposed gBOINS design unifies the continuous and quasi-binary toxicity endpoints as well as the standard binary endpoint. Different from the gBOIN design, the decision boundaries of gBOINS design, $\lambda _{e}^{*}(n_{j})$ and $\lambda _{d}^{*}(n_{j})$ were adaptive, which provides the statistician a flexible tool to control the over-toxicities. The design can also converge to the target toxicity probability as the sample size goes to infinity. This unique convergence property of gBOINS was demonstrated both theoretically and numerically. Compared to the gBOIN design, when there were more than one doses lying inside the decision boundaries $\left (\lambda _{e}^{*}, \lambda _{d}^{*}\right)$ determined by the gBOIN design, the gBOINS had a substantial improvement in terms of the PCS when the sample size was moderate large. Also, we showed that when the sample size was small, the performance of gBOINS design was comparable with the gBOIN design in terms of the PCS and can allocate more patients to safe doses by simulations.

Although the prosed gBOINS design focus on phase I trial designs, similarly to the BOIN-ET proposed by [28], it can be directly extended to the phase I/II designs. One limitation of the gBOINS design is that it assumes toxicity outcome can be observed quickly enough to make the dose assignment decisions for each enrolled cohort. One approach to extend the gBOINS design to accommodate late-onset or delayed outcomes, for example, would be to use the Bayesian data augmentation approach [29, 30] or the approximated likelihood approach [31]. This is a topic of our future research.

## Data Availability

Not applicable.
